# Identification of *Trypanosoma cruzi* Discrete Typing Units (DTUs) through the implementation of a High-Resolution Melting (HRM) genotyping assay

**DOI:** 10.1186/1756-3305-6-112

**Published:** 2013-04-20

**Authors:** Sonia L Higuera, Felipe Guhl, Juan David Ramírez

**Affiliations:** 1 Centro de Investigaciones en Microbiología y Parasitología Tropical, CIMPAT, Universidad de Los Andes, Cra 1ª No. 18ª-10, Bogotá, Colombia

**Keywords:** Chagas disease, *Trypanosoma cruzi*, DTU, high-resolution melting, Genotyping

## Abstract

**Background:**

Chagas disease, caused by *Trypanosoma cruzi,* is a geographically widespread anthropozoonosis that is considered a major public health problem in Latin America. Because this parasite presents high genetic variability, a nomenclature has been adopted to classify the parasite into six discrete typing units (DTUs): TcI, TcII, TcIII, TcIV, TcV, and TcVI, which present different eco-epidemiological, clinical, and geographic associations. Currently, the available genotyping methods present a series of drawbacks that implies the need for developing new methods for characterizing *T. cruzi* DTU’s. The aim of this work was to genotype reference populations from *T. cruzi* by means of a High-Resolution Melting (HRM) genotyping assay.

To genotype the DTUs of 38 strains and 14 reference clones of *T. cruzi* from diverse sources, real-time PCR (qPCR) was coupled to high-resolution melting (HRM) based on the amplification of two molecular markers—the divergent domain of the 24 sα rRNA gene and the intergenic region of the mini-exon gene.

**Findings:**

Amplification of the mini-exon gene allowed the genotyping of three distinct groups: TcI, TcII- TcIV- TcV, and TcIII-TcVI, while amplification of the 24sα gene generated non-overlapping melting temperature ranges for each DTU that were used to identify the groups in the six existing DTUs of *Trypanosoma cruzi*.

**Conclusions:**

The proposed genotyping assay allowed discrimination of the six genetic groups by obtaining specific melting curves for each DTU. The application of this technique is proposed because of its specificity, sensitivity, high performance, and low cost compared with other previously described characterization methods.

## Findings

Chagas disease is an anthropozoonosis caused by the parasite *Trypanosoma cruzi* and affects an estimated 10 million people, posing a serious public health problem [1]. *Trypanosoma cruzi* has a high genetic variability with evidence of six Discrete Typing Units (DTU’s): *T. cruzi* I (TcI), *T. cruzi* II (TcII), *T. cruzi* III (TcIII), *T. cruzi* IV (TcIV), *T. cruzi* V (TcV), and *T. cruzi* VI (TcVI), each of which presents characteristics based on the geographic distribution, eco-epidemiological associations, and clinical manifestations of the disease [2]*.* Currently, several methods have been reported for characterizing and genotyping *Trypanosoma cruzi* populations, including the PCR amplification of the SL-IR (spliced-leader intergenic region of the mini-exon gene), 24sα, and 18s rDNA gene regions. The comparison criterion for this technique is based on the size differences in the resulting amplicons [3,4]. However, the characterization obtained using this technique is limited by the absence of amplification products or the need for multiple amplification procedures [2,5]. Another proposed methodology is based on genetic polymorphism analysis using multilocus PCR-RFLP; however, this technique requires the development of combinations and multiple assays, increasing its complexity and limiting its use [6,7]. In 2009, Llewellyn and colleagues implemented an approach based on the Multilocus Microsatellite Typing (MLMT) of a panel of 48 polymorphic microsatellite markers. This technique is a quick and feasible alternative but requires the inclusion of sequencing procedures that lead to increased costs [8]. A similar initiative was proposed to develop a Multilocus Sequence Typing (MLST) scheme based on the analysis of a panel of four molecular targets: Met-III, RB19, TcGPXII, and DHFR-TS; however, the development of this process requires both bioinformatic analysis and sequencing steps [9]. Recently, the analysis of the polymorphisms of the 24sα rDNA gene, the subunit II of the cytochrome oxidase, and four microsatellite loci has been shown to be an alternative method [10]. This technique is highly efficient but requires multiple amplification steps that can lead to an increased risk of contamination.

Thus, a low-cost genotyping technique with reliable and robust results for the identification of the six genetic groups in a single assay is needed to facilitate the characterization of the circulating strains in a specific geographic area. A technique such as High-Resolution Melting (HRM) offers a viable alternative for genotyping *T. cruzi* given its robustness, speed, cost, and the elimination of sequencing or hybridization procedures [11]*.* This molecular analysis has recently been used to genotype different populations of parasitic microorganisms [12-15]. The aim of this study was to genotype 36 reference strains and 18 reference clones of *Trypanosoma cruzi* previously genotyped by different molecular markers from different biological and geographical origins via HRM.

We employed 36 strains and 18 reference clones of *Trypanosoma cruzi* previously characterized via MLST, MLEE, MLMT, and RFLP-PCR (Table 1). Additionally, in order to test specificity we included DNA from reference strains of *T. rangeli* and *Leishmania panamensis*. A real-time PCR assay coupled with HRM analysis was developed to target SL-IR and the 24sα rRNA gene using a 7500 Fast Real-Time PCR System (Applied Biosystems, Inc., CA, USA). These genes were selected because they had previously been used as molecular markers for the PCR genotyping of *T. cruzi*[4,16]. The amplification reactions were carried out in a total volume of 21 μL. The reaction mixture consisted of 1× MeltDoctor™ HRM Master Mix (Applied Biosystems, Inc., CA, USA); a 5 μM solution of each of the primers TCC (5^′^CCC CCC TCC CAG GCC ACA CTG 3) and TC2 (5^′^CCT GCA GGC ACA cGT GTG TGT G 3) for the amplification of SL-IR and 24sα, D71 (5^′^AAG GTG CGT CGA CAG TGT GG 3) and D72 (5^′^TTT TCA GAA TGG CCG AAC AGT 3); 6.6 μL of water; and 20 ng/μL DNA template. The cycle conditions for the qPCR were as follows: 95°C for 10 minutes (1 cycle), followed by amplification for 40 cycles at 95°C for 15 seconds (denaturation) and 60°C for 1 minute. The amplicon dissociation was immediately initiated by a melting step. The thermal profile consisted of denaturation at 95°C for 10 seconds, 60°C for 1 minute (annealing), 95°C for 30 seconds (high-resolution melting), and a final annealing stage at 60°C for 15 seconds. The melting curve analysis was carried out by supplying raw fluorescence (F) vs. temperature (T) values or already transformed -dF/dT values. The raw value,differentiation was performed and the first derivative curve is displayed with the melting peaks. Peaks and Tm values were automatically identified, peak areas calculated and peaks flagged as ‘negative’ if the peak area is below the user-defined threshold value. During this process, the PCR amplicons were denatured prior to developing the melting curves, and the changes in fluorescence with respect to the changes in temperature (dF/dT) were recorded by the instrument with a ramp of 0.5°C/seg. Each DNA sample was analyzed in duplicate. Finally, the normalized and derivative melting curve profiles were obtained. The analysis was performed using a blind methodology to reduce the possibility of bias. To evaluate the reproducibility of the assay, the genotyping technique was performed on five independent days under the same conditions. The specificity was assessed by evaluating the concordance between the pre-established genotypes of the DNA samples used and the genotypes characterized by HRM. The consistency of the method was determined by correlating the previously quantified reproducibility and specificity [17]. Finally, a randomization and subsequent non-parametric Kruskal Wallis test (p < 0.05) were conducted to detect any significant differences between the melting temperature data for each DTU. The analyses were performed using the R platform and GraphPad software package.

**Table 1:** **Biological and geographical origin of the 36 reference strains and 18 reference clones of *Trypanosoma cruzi *previously genotyped by MLEE, MLST and MLMT employed in the HRM analyses**

**DTU**	**Strain/clone**	**Geographical origin**	**Biological origin**
TcI	X10610	Guárico, Venezuela	*Homo sapiens*
	92101601	Georgia, USA	*Didelphis marsupialis*
	Cutia cl1*	Espiritu Santo, Brazil	*Dasyprocta aguti*
	DM28	Carabobo, Venezuela	*Didelphis marsupialis*
	Jr Cl4*	Anzoátegui, Venezuela	*Homo sapiens*
	x101	Pará, Brazil	*Homo sapiens*
	Saxp18	Maje, Perú	*Homo sapiens*
	Chilec22*	Flor de Valle, Chile	*Triatoma spinolai*
	10r 26	Santa Cruz, Bolivia	*Aotus sp.*
	c8	La Paz, Bolivia	*Triatoma infestans*
	ERA	Anzoátegui, Venezuela	*Homo sapiens*
	SP104cl1*	Región IV, Chile	*Mepraia spinolai*
	P209cl93*	Sucre, Bolivia	*Homo sapiens*
	OPS21cl11*	Cojedes, Venezuela	*Homo sapiens*
TcII	MAScl1*	Minas Gerais, Brazil	*Homo sapiens*
	IVVcl4*	Cuncumen, Chile	*Homo sapiens*
	Pot 7b	San Martin, Paraguay	*Triatoma infestans*
	CBB	Tulahuen, Chile	*Homo sapiens*
	Pot 7a Cl*	San Martin, Paraguay	*Triatoma infestans*
	Chaco 23	Chaco, Paraguay	*Triatoma infestans*
	EBcl21*	Boyacá, Colombia	*Homo sapiens*
	Tu18	Tupiza, Bolivia	*Triatoma infestans*
	ESM	Sao Felipe, Brazil	*Homo sapiens*
TcIII	85/847	Alto Beni, Bolivia	*Dasypus novemcinctus*
	M6421	Belem, Brazil	*Homo sapiens*
	SLDN1	Casanare, Colombia	*Dasypus novemcintus*
	Ua2	Sucre, Bolivia	*Dasypus novemcintus*
	SABPI9	Vitor, Perú	*Triatoma infestans*
	M5631cl5*	Marajo, Brazil	*Dasypus novemcinctus*
TcIV	Stc10r	Georgia, USA	*Procyon lotor*
	92122	Georgia, USA	*Procyon lotor*
	CANIII	Belem, Brazil	*Homo sapiens*
	TV	Boyacá, Colombia	*Triatoma venosa*
	DOG THEIS	Oklahoma, USA	*Canis familiaris*
TcV	92.80	Santa Cruz, Bolivia	*Homo sapiens*
	Chaco 9	Chaco, Paraguay	*Triatoma infestans*
	Para6	Chaco, Paraguay	*Triatoma infestans*
	PPAH179 CL5*	Chaco, Argentina	*Homo sapiens*
	Chaco 2 CB	Chaco, Paraguay	*Triatoma infestans*
	AACF2	Casanare, Colombia	*Canis familiaris*
	Para4	Paraguari, Paraguay	*Triatoma infestans*
	sc43	Santa Cruz, Bolívia	*Triatoma infestans*
	Vinch101	Limarí, Chile	*Triatoma infestans*
	MN cl2*	Region IV, Chile	*Homo sapiens*
	BUG2148cl1*	Rio Grande do Sul, Brazil	*Triatoma infestans*
	SC43cl1*	Santa Cruz, Bolivia	*Triatoma infestans*
	SO3cl5*	Potosi, Bolivia	*Triatoma infestans*
TcVI	LhVa	Chaco, Argentina	*Triatoma infestans*
	VfrA	Francia, Chile	*Triatoma infestans*
	Tula Cl2*	Tulahuen, Chile	*Homo sapiens*
	Rp540	Casanare, Colombia	*Rhodnius prolixus*
	p251	Cochabamba, Bolivia	*Homo sapiens*
	CLBRENER	Rio Grande do Sul, Brazil	*Triatoma infestans*
	P63cl1*	Makthlawaiya, Paraguay	*Triatoma infestans*

*Biological Clones.

**Figure 1:**
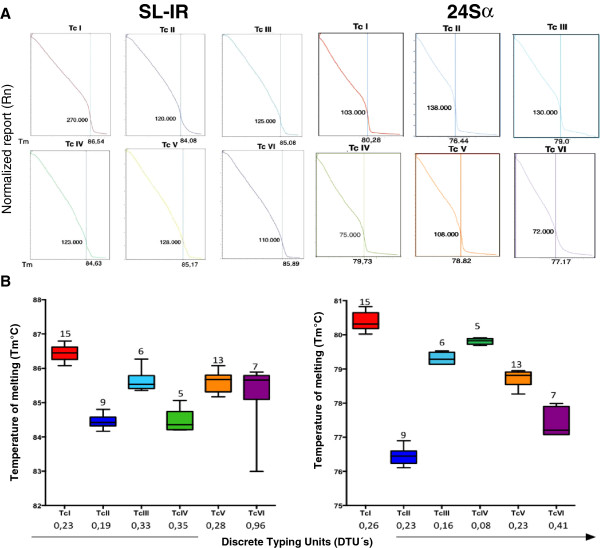
**Melting Temperature profiles and scatter plots of the HRM genotyping assay. A**. Representative Normalized melting curves of the *Trypanosoma cruzi* Discrete Typing Units **B**. Scatter-plot showing overlapping and non-overlapping changes in the Tm values of the curves obtained by each DTU; the values above the boxes indicate the number of strains/clones employed per DTU.

**Figure 2:**
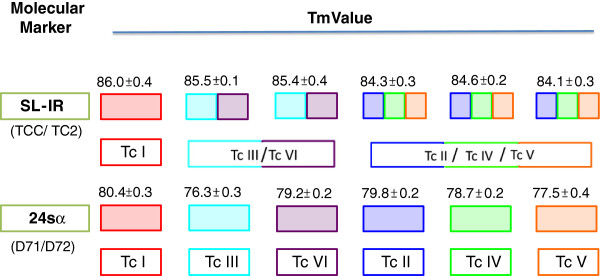
**Feasible and accurate algorithm showing the use of HRM assay for the discrimination of *Trypanosoma cruzi *DTU’s.**

A specific melting temperature was obtained for each of the reference strains and clones included in this study, both for amplifying SL-IR and for the 24sα. The plots were created for the melting temperature distribution of each DTU (Figure 1). The 24sα gene exhibited higher discriminatory power than the SL-IR gene, producing non-overlapping ranges of the melting temperature for each DTU. The normalized fluorescence curve and the derivative melting curve yielded a different and easily distinguishable profile for each DTU. The representative profiles were collected for the derivative, the normalized melting curves for the SL-IR gene and the 24sα amplicons (Figure 1). Finally, an algorithm was constructed (Figure 2) which allowed the discrimination of the DTU’s after the amplification of the SL-IR gene and the subsequent amplification of the 24sα gene. To validate the method, three parameters were determined based on the methodology of Moncayo and Luquetti (1990) [17]; the reproducibility was determined for both the SL-IR gene amplification and the 24sα rRNA and yielded a value of 100% for both tests. The specificity and consistency values were obtained only for the amplification of the 24sα rRNA, which was the only marker capable of generating non-overlapping melting temperature ranges that was specific for the six DTU’s and the DNAs from *T. rangeli* and *L. panamensis*.

The use of the HRM methodology allowed the adequate and congruent discrimination of the *T. cruzi* genotypes for the strains and reference clones used. Many problems, such as the need for sequencing, the degree of complexity, the design of multiple pairs of primers, and genotyping by the absence of a band, among others, have been reported with the existing genotyping methods. Thus, new alternatives are needed to simplify the process and minimize the difficulties. For example, methods based on the absence of an amplification product are considered problematic because no actual evidence is generated that may relate to the presence of a specific DTU [2]. To obtain a correct identification, other genotyping methods for which high efficiency, sensitivity, and robustness have been reported require multiple amplifications of a single sample using different markers or coupled with RAPD’s and RFLP analysis, which can significantly increase the economic cost and lead to sample contamination [18]. The HRM technique does not present any of the aforementioned difficulties and, given the as-obtained results, could be considered a potential alternative strategy for genotyping *T. cruzi*.

The markers were selected based on two criteria: the fact that both the SL-IR gene and the 24sα rRNA have previously been used to characterize *T. cruzi* and because they exhibit a high number of copies within the parasite genome, which increases the probability of detection [6,19,20]. The results of this study indicated that the SL-IR gene did not allow the absolute discrimination of the six DTUs in a single assay, a fact that may be due to the recently confirmed variation in the copy number of the gene and the chromosomal rearrangement at the level of the different strains [21]. To examine the reproducibility of the method, the assays were conducted in duplicate, and the tests were repeated over several days, maintaining the same test conditions, reagent quantities, and DNA concentrations. The technique generated a reproducibility value of 100% for both molecular markers. This result is of great significance because genotyping techniques must be reproducible and robust to avoid producing conflicting results and to accurately establish the genotype of each sample analyzed [22].

## Conclusion

The use of a real-time PCR assay coupled with HRM analysis is proposed for genotyping the DTU’s of the *T. cruzi* population for the development of research projects that require genotyping of the circulating strains because it provides an easy, quick, and low (1.5 USD per assay compared to 2.5 USD for genotyping) alternative compared to the existing methods. Among the advantages over other currently used methods are its speed and simplicity (given that this method does not require gel electrophoresis), the reduction of the processing time, and the specificity and sensitivity of the method [14]. Therefore, HRM coupled to qPCR provides a cost-effective alternative for the future genotyping and identification of the six DTU’s of *T. cruzi* which fulfills one of the main research targets in Chagas disease*.*

## Abbreviations

DTU: Discrete Typing Unit; TcI: *Trypanosoma cruzi* I; TcII: *Trypanosoma cruzi* II; TcIII: *Trypanosoma cruzi* III; TcIV: *Trypanosoma cruzi* IV; TcV: *Trypanosoma cruzi* V; TcVI: *Trypanosoma cruzi* VI; HRM: High-Resolution Melting; SL-IR: Spliced Leader Intergenic Region.

## Competing interests

The authors declare that they have no competing interest.

## Authors’ contributions

JDR designed the experiments. SLH and JDR standardized the HRM assay and developed the experiments to calculate the parameters of reproducibility and specificity. SLH, JDR and FG wrote the manuscript. All authors read and approved the final version of the manuscript.
